# Energy expenditure and affect responses to different types of active video game and exercise

**DOI:** 10.1371/journal.pone.0176213

**Published:** 2017-05-01

**Authors:** Javier Monedero, Enda E. Murphy, Donal J. O’Gorman

**Affiliations:** School of Health and Human Performance, Faculty of Science and Health, Dublin City University, Dublin, Ireland; Sao Paulo State University, BRAZIL

## Abstract

**Background:**

The purpose of this study was to compare entertainment-themed active video game (AVG) and fitness-themed AVG play with traditional exercise to examine the interaction between physiological and psychological responses.

**Methods:**

Participants (N = 23) were randomly assigned to 30-min of (i) self-selected intensity exercise (SS-EX), (ii) moderate intensity exercise (MOD-EX), (iii) entertainment-themed video game (ET-VG) and (iv) fitness-themed video game (FT-VG). Physiological and psychological outcomes were recorded before, during and after each trial.

**Results:**

All trials met the ACSM criteria for moderate or vigorous physical activity. The $\%\dot{V}O_{2}R$ (68.3±13.9%) and rate of energy expenditure (10.3±3.1kcal/min) was significantly higher in the SS-EX trial with lowest values reported for ET-VG (p<0.05). No differences were found in % heart rate reserve between SS-EX and FT-VG (66.9±12.5% and 67.1±6% respectively). The AVG’s were significantly more enjoyable than the exercise trials (p<0.05) and the ET-VG resulted in the highest core flow and psychological well-being (p<0.05).

**Conclusion:**

AVG’s can elicit physiological responses that meet recommended exercise intensities but are more enjoyable than conventional exercise in young inactive adults. While further work is required, this study highlights the importance of examining the interaction between physiological outcomes and psychological states to increase physical activity and reduce sedentary time.

## Introduction

Physical inactivity and sedentary behaviour are independent predictors of all-cause mortality and the development of non-communicable diseases[1]. Physical inactivity was responsible for 3.2 million deaths and 2.8% of disability adjusted life years (DALYs) in 2010 and was tenth on the list of global health risks[2]. Similarly, sedentary behaviour, defined as an energy expenditure ≤1.5 METs while sitting or lying [3], is associated with increased risk of diabetes, cardiovascular disease and all-cause mortality[1]. One of the major societal challenges is to increase physical activity levels and the best health outcomes may be achieved by substituting sedentary time.

The determinants of physical activity participation and adherence are multifactorial and complex[4]. A better understanding of the interaction between psychological determinants and physiological outcomes is necessary to design more innovative and effective strategies to increase physical activity. Affective judgements and perception of effort during an acute exercise session are determinants of intention and future exercise behaviour [5, 6]. Affective judgements refer to self-reported measures of enjoyment, pleasure or displeasure and feeling state that results from engaging in physical activity. Williams *et al*. [7] showed that affective judgements to moderate intensity exercise (feeling scale score) was a good predictor of exercise behaviour 6 to 12 months later. Also, Vazou-Ekkekais suggests that “the optimization of affective response should be taken into account when recommending or prescribing PA to the public”[8]. In addition, an individual’s perception of effort is, to a certain extent, a determining factor of exercise adherence[9].

One of the most reported affective judgements in the literature is enjoyment. According to Wankel[10], enjoyment plays a central role in the promotion of exercise adherence and psychological well-being. Negative affective judgements can be a contributing factor to the 40–65% of dropout rates within the first 3–6 months of commencing an exercise programme[11, 12]. An exercise prescription that leads to health enhancement and positive affective judgements is most likely to result in high adherence rates. In the context of affective judgements related to physical activity, the dual-mode model theory by Ekkekakis needs to be considered[13, 14]. This theory suggest that affective responses to exercise are influenced by the continuous interplay of cortically mediated cognitive processes (e.g., self-efficacy, self-presentational concerns, goals, attributions) and ascending interoceptive cues (e.g., ventilation, acidosis, core temperature). Positive affect states decrease as the ventilatory threshold (VT) stage is reached and passed.

There is potential for a relatively new technology, active video games (AVGs) to have a tangible impact on physical activity and reducing sedentary time while maintaining or enhancing enjoyment. Active video games require the player to get off the couch and move to be able to play. Approximately 42% of Americans play video games regularly or at least 3 hours per week [15]. In addition to this, the average age of the most frequent purchaser is 35 years old. There is strong evidence that AVGs result in higher heart rate (HR), oxygen consumption ($\dot{V}O_{2}$) and energy expenditure (EE) in adults and children[16–18] than traditional sedentary video game play. Also, a growing number of studies have shown that AVGs elicit light-to-vigorous intensity exercise and some have suggested that they could be a contributor to health enhancing physical activity[18–23]. There has been a proliferation of fitness and entertainment-themed AVGs in the market over the last few years. While there is some data on the physiological stimulus and affective judgements associated with playing different types of AVG, there are a lot of unknown aspects. Affective judgments like flow or psychological well-being while playing different types of AVG play has not been studied. The state of flow represents a condition of supreme enjoyment in which the participant is immersed in the task at hand.

The aim of this study is to compare the physiological responses and affective judgements during conventional exercise and while playing AVGs (fitness-themed and entertainment-themed). Our hypothesis is that the AVGs will result in similar rates of energy expenditure and more positive affective judgements than conventional moderate exercise intensity and that AVGs provide an adequate stimulus to meet the PA guidelines for healthy adults.

## Materials and methods

### Participants

Twenty-three young, healthy men and women volunteered to take part in this study. Participants were excluded if they were >45 yrs, smoked cigarettes, performed >1 exercise session per week in the previous 6 months or had a $\dot{V}O_{2}\max$ in the top 40^th^ percentile for their age and sex. Participants completed a Physical Activity Readiness Questionnaire for Everyone (PAR-Q+)[24], and a general health questionnaire. The study was approved by the Dublin City University Research Ethics Committee (DCUREC/2011/071), complied with the Declaration of Helsinki (2013) and all participants provided written informed consent.

### Experimental overview

**Fig 1:**
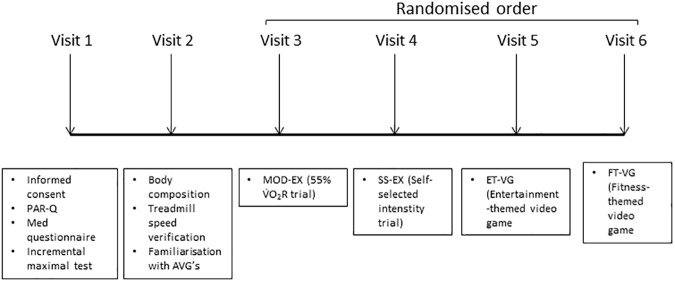
Schematic of study protocol.

Participants reported to the laboratory on 6 days separated by at least 48 h (see Fig 1). They were asked to refrain from consuming alcohol for at least the 24 h prior to each visit and from the ingestion of food, caffeinated products and tea for 3 h before each visit. The first two visits were used to measure maximal aerobic capacity, to verify the exercise intensity and to familiarize participants with the AVGs. After this, participants reported to the laboratory where they performed 4 trials using a randomized, cross-over design. The order of trials was determined using an online randomisation tool[25]. The participants remained seated for 10 min prior to and for 20 min after completing 30 min of (i) running at a self-selected exercise intensity (SS-EX); (ii) running at 55% $\dot{V}O_{2}\mathrm{Reserve}$ (MOD-EX); (iii) playing an entertainment themed video game (ET-VG) and (iv) playing a fitness themed video game (FT-VG).

An incremental maximal test was used to determine $\dot{V}O_{2}$ max using open-circuit spirometry (K4B2, Cosmed, Rome, Italy). Participants were also fitted with a heart rate monitor (Polar RS400, Polar, Oulu, Finland). The protocol consisted of a 3 min warm-up followed by 2 min incremental stages until the participant reached volitional fatigue. The $\dot{V}O_{2}$ max was considered to have been reached using ACSM guidelines[26]. On a separate occasion, the speed corresponding to 55% $\dot{V}O_{2}R$ was determined for the MOD-EX trial. Participants ran for 10 min at three different speeds: 1 km/h below the predicted speed, at the predicted speed and 1 km/h above the predicted speed. The $\%\dot{V}O_{2}R$ was determined by using the linear function, *y = mx+c*, where *y* is $\dot{V}O_{2}$, *x* is treadmill speed, *m* is the slope of the relationship between $\dot{V}O_{2}R$ and treadmill speed and *c* is the y-axis intercept.

The active video game trials were played using XBOX 360 (Microsoft Corp, Redmond, USA). This platform was chosen because it offers a wide range of entertainment and fitness-themed video games. The ET-VG trial used Kinect Adventures (Microsoft Corp, Redmond, USA) while the FT-VG used Your Shape Fitness Evolve (Ubisoft, Surrey, UK). A summary of the games and the number of times they were played is outlined in the supporting information file (S1 Appendix). Immediately after completing the 30 min run or play, participants sat down and filled in the psychological questionnaires.

In the SS-EX trial, participants were told that they could select the speed of the treadmill for the first minute of every 5 min block while the gradient was kept constant at 1%. Participants were instructed to select an intensity that they would be able to maintain for 30-min and were made aware that they could alter the speed every 5 min. In the MOD-EX trial, participants exercised at a constant speed that was determined during the familiarization session. The intensity of 55% $\dot{V}O_{2}\mathrm{Reserve}$ was selected because it corresponds to moderate intensity exercise and it was below the ventilatory threshold in a similar cohort to the one used in this study (unpublished data).

Baseline measures were obtained by averaging the last 3 min of the 10 min data collection at rest prior to each trial. During trials data was averaged for the entire 30-min trial and in two 15-min segments. The rate of energy expenditure was estimated using the following formula as calculated by the Cosmed K4B2 using the abbreviated Weir equation assuming urea nitrogen was zero[27]:

$$\begin{array}{l} EE\;\left(Kcal\cdot min^{-1}\right)=\left(3.781*\;\dot{V}O_{2}\right)+(1.237*\dot{V}CO_{2})\;if\;urea\;nitrogen=0 \end{array}$$ (1)

Heart rate reserve and $\dot{V}O_{2}$ reserve ($\dot{V}O_{2}R$) were calculated using standardised equations[26]. Individual ventilatory threshold (VT) was calculated by the function available in the Cosmed K4B2 software, which is based on the V-slope method[28]. The % $\dot{V}O_{2}\max$ above or below VT during the trials was calculated using the following formula:

$$\%VT=\left(\left(\dot{V}O_{2}trial\;-\dot{V}O_{2}@VT\right)\;x\;100\right)\;/\dot{V}O_{2}@VT$$ (2)

where % VT = % $\dot{V}O_{2}\max$ above or below the $\dot{V}O_{2}@$ VT, $\dot{V}O_{2}\mathrm{trial}$ = average oxygen uptake during the trial in L/min, $\dot{V}O_{2}@\mathrm{VT}$ = oxygen uptake at VT in L/min.

The interest/enjoyment subscale of the Intrinsic Motivation Inventory was used to measure enjoyment. This scale has been used in other AVG [29] and exercise studies [30] and has been found to be a valid and reliable measure. Participants ranked their agreement with each statement on a 7-point Likert scale during and immediately after each trial. Responses were averaged to create the enjoyment score (range = 1–7). At 15 min, participants were asked to step off the treadmill or stop playing while they filled in the scale.

The State of Flow represents a condition of supreme enjoyment in which the individual is immersed in the task. Participants filled in the Core Flow State Scale (CFSS) to measure the state of flow [31]. The CFSS contains 10 items that are descriptions of what it feels like to be immersed in an activity. This scale is designed to be used as a post-event flow assessment therefore responses were provided immediately after completing each trial.

We used the 15 point Borg Rate of perceived exertion (RPE scale) to determine participants’ level of exertion at min 15 and 29 during each trial.

Psychological well-being was measured with the Subjective Exercise Experience Scale (SEES). This scale has been used in previous exercise studies [32] and video games studies [33]. This is a 12-item questionnaire that is scored on a 7-point Likert- scale. The SEES provides three subscales: 1) psychological well-being (great, positive, strong, terrific); 2) psychological distress (awful, crummy, discouraged, miserable); and 3) fatigue (drained, exhausted, fatigued, tired). Though the SEES is a reliable tool for psychological well-being there are some conceptual and methodological criticisms[34]. We measured psychological distress and fatigue but we are not reporting the data as the internal validity was not acceptable.

### Statistical analysis

All tabular values are reported as the mean ± SEM and significance set at p<0.05. All data were tested for normal distribution (Shapiro-Wilk test) and the Greenhouse Geisser correction for degrees of freedom was applied when sphericity was not met. To investigate differences within subjects by trial and by time, we performed a mixed design ANOVA with sex as a between-subjects variable. Multiple comparison tests were computed using the Bonferroni correction. Unpaired t-tests were used to detect significant differences in subject characteristics. A Spearman correlation was used to assess the relation between enjoyment and ventilatory threshold.

## Results

### Participant characteristics

As expected, the men were heavier, had a lower body fat and a higher aerobic capacity when compared to the females. They were also older than the females but had a similar BMI, HRrest and HRmax. The physical characteristics of the participants are summarised in Table 1.

**Table 1:** Physical characteristics and cardiorespiratory responses during $\dot{V}O_{2}$ max test.

	Male (n = 11)	Female (n = 12)	All (n = 23)
**Age (yrs)**	26.8±1^a^	22.9±1	24.8±1
**Height (m)**	1.78±0.08^a^	1.65±0.05	1.70±0.09
**Weight (kg)**	84.8±4.6^a^	69.1±2.6	76.6±3
**BMI (kg.m**^**-2**^**)**	26.4±1.2	25.3±0.9	24.8±1.3
**Body fat (%)**	19.1±1.9^a^	32.2±1.4	25.9±1.8
**HRrest (beats**^**.**^**min**^**-1**^**)**	60±3	62±2	61±2
**HRmax (beats**^**.**^**min**^**-1**^**)**	198±2.1	193.8±2.1	195.8±1.5
$\dot{V}O_{2}\mathrm{rest}$ **(L**^**.**^**min**^**-1**^**)**	0.314±0.050	0.261±0.026	0.291±0.050
$\dot{V}O_{2}\mathrm{rest}$**(mL**^**.**^**kg**^**-1.**^**min**^**-1**^**)**	4.1±0.1	3.8±0.2	3.8±0.1
$\dot{V}O_{2}\max$**(L**^**.**^**min**^**-1**^**)**	3.3±0.2^a^	2.4±0.1	2.9±0.1
$\dot{V}O_{2}\max$ **(mL**^**.**^**kg**^**-1.**^**min**^**-1**^**)%**	40.3±1.2^a^	35.8±1.1	37.9±0.9
$\%\dot{V}O_{2}\max$ **@ VT**	70.3±2	71.5±2.1	70.9±1.5

Data presented as Mean ± SEM.

^a^ significantly different to females (p <0.05). HRrest: heart rate at rest, HRmax: maximal heart rate, $\dot{V}O_{2}\max$: maximal oxygen uptake. VT: ventilatory threshold.

There were no significant differences in baseline $\dot{V}O_{2}$, HR and energy expenditure before the four exercise or game trials.

### Physiological responses

All trials reached the criteria for moderate or vigorous intensity PA. The $\%\;\dot{V}O_{2}\max$ was significantly higher during the SS-EX trial than MOD-EX, ET-VG trial (*P* < 0.001) and FT-VG. (p < 0.005), partial η^2^ = 0.823. The % $\dot{V}O_{2}\max$ was similar during MOD-EX and FT-VG and significantly higher than ET-VG (Table 2). $\%\dot{V}O_{2}\max$ increased over time during all trials *P* < 0.001) partial η^2^ = 0.695) and was greater in males than females (p < 0.005), η^2^ = 0.351.

**Table 2:** Cardio-metabolic response to exercise and AVG trials.

	SS-EX	MOD-EX	ET-VG	FT-VG
**Average HR (bpm)**	151.2±3.6^b^	129.0±3.3	119.4±6.3	151.6±2.1^b^
**%HRmax**	77.3±1.9^b^	65.9±1.5	60.9±3.1	77.4±0.8^b^
**%HRR**	66.9±2.7^b^	50.4±2.1	46.5±4.7	67.1±1.2^b^
**Average** $\dot{V}O_{2}$ **(mL**^**-1.**^**kg**^**-1.**^**min**^**-1**^**)**	27.4±1.3^a^	21.7±0.7	17.9±0.9^a^	22.2±0.8
**%** $\dot{V}O_{2}\max$	71.6±2.8^a^	56.9±0.9	46.9±1.6^a^	58.6±1.6
**%** $\dot{V}O_{2}R$	68.3±3.1^a^	52.2±1	41.1±1.8^a^	53.9±1.8
**Rate of EE (kcal**^**.**^**min**^**-1**^**)**	10.3±0.7^a^	8.1±0.4	6.7±0.4^a^	8.5±0.4
**MET**	7.8±0.4^a^	6.2±0.2	5.1±0.3^a^	6.4±0.2
**%VT**	-1.73±0.62^a^	-19.71±0.46	-34.22±0.57^a^	-18.24±0.63

Data presented as mean ± SEM. EE: energy expenditure (kcal^.^min^-1^); HR: heart rate; HRR: heart rate reserve; MET: metabolic equivalent of the task (3.5 mL^.^kg^-1.^min^-1^); %VT: % $\dot{V}O_{2}\max$ above or below the ventilatory threshold. Significant differences are shown only for statistical comparison of total data.

^a^ significantly different to all other trials(p < 0.05).

^b^ significantly different than MOD-EX and ET-VG (p < 0.05).

A significant effect of trial (p < 0.001), partial η^2^ = 0.897 and time (p < 0.001), partial η^2^ = .872 was found for heart rate (Table 2). There were no differences in % HRmax between the SS-EX trial and the FT-VG and both were significantly greater than the MOD-EX and ET-VG trials (p < 0.001). Percentage HRmax increased over time across all trials (p < 0.001) except during MOD-EX as expected with a greater increase in SS-EX and ET-VG. We did not find any gender effect in the HR response to the different conditions.

**Fig 2:**
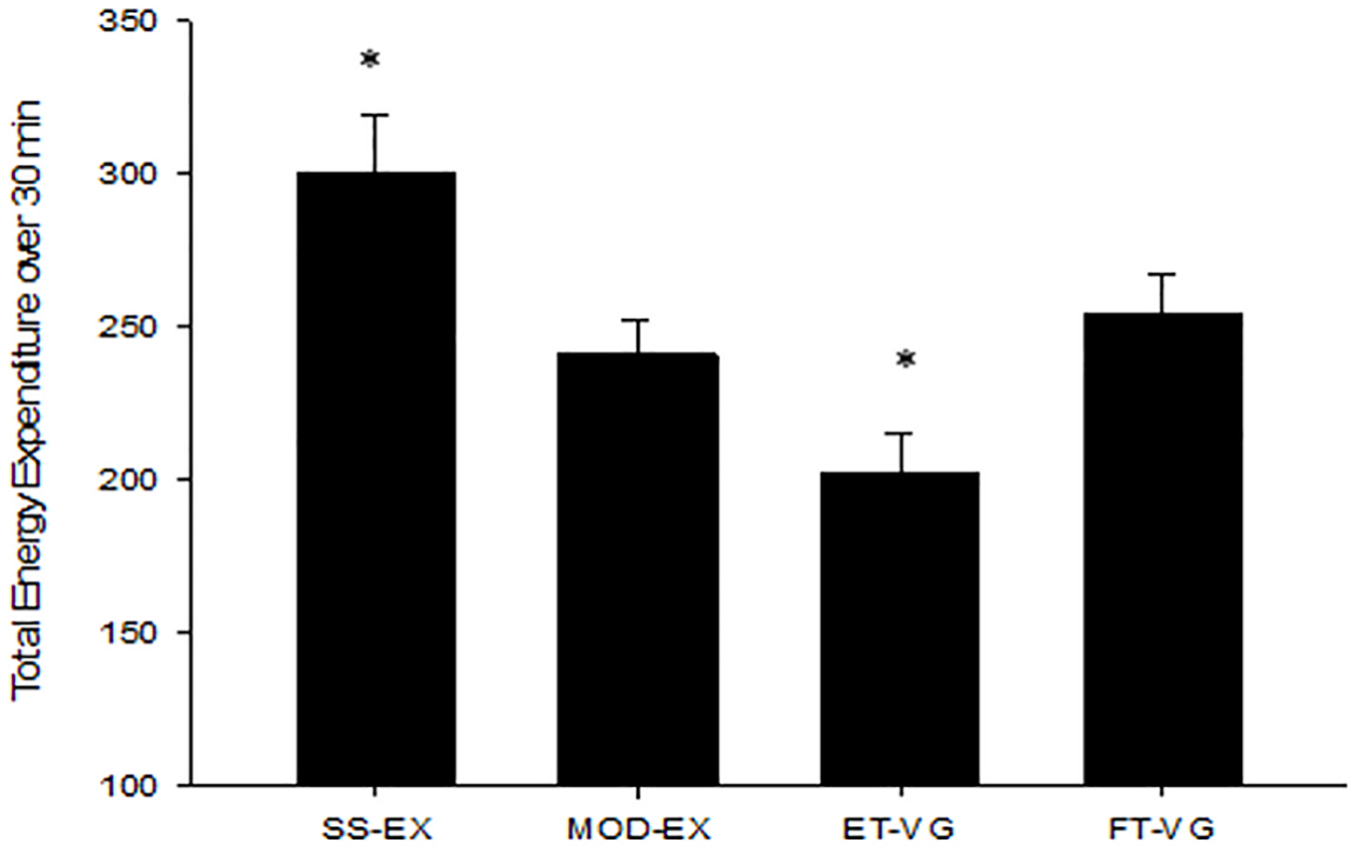
Total amount of EE (kcal) in the 4 different trials. All data presented as mean ± SEM. *significantly different to all other trials (*P* <0.05).

The SS-EX trial resulted in a significantly higher rate of energy expenditure than MOD-EX (p < 0.001), ET-VG (p < 0.001) and FT-VG (p< 0.005), partial η^2^ = 0.801. No significant differences were found between MOD-EX and FT-VG. Both MOD-EX and FT-VG resulted in significantly higher rates of EE than ET-VG (p < 0.001). The rate of energy expenditure increased significantly over time during the SS-EX and ET-VG trials (p < 0.001), η^2^ = 0.721 but not during MOD-EX and FT-VG trials. A between subjects effect of gender (*P* < 0.001) was also detected, indicating than males had a significantly higher rate EE than females. As results of the different rates of EE in the different trials, there were significant differences in the total amount of EE as Fig 2 shows.

### Affective and perceptual responses

**Fig 3:**
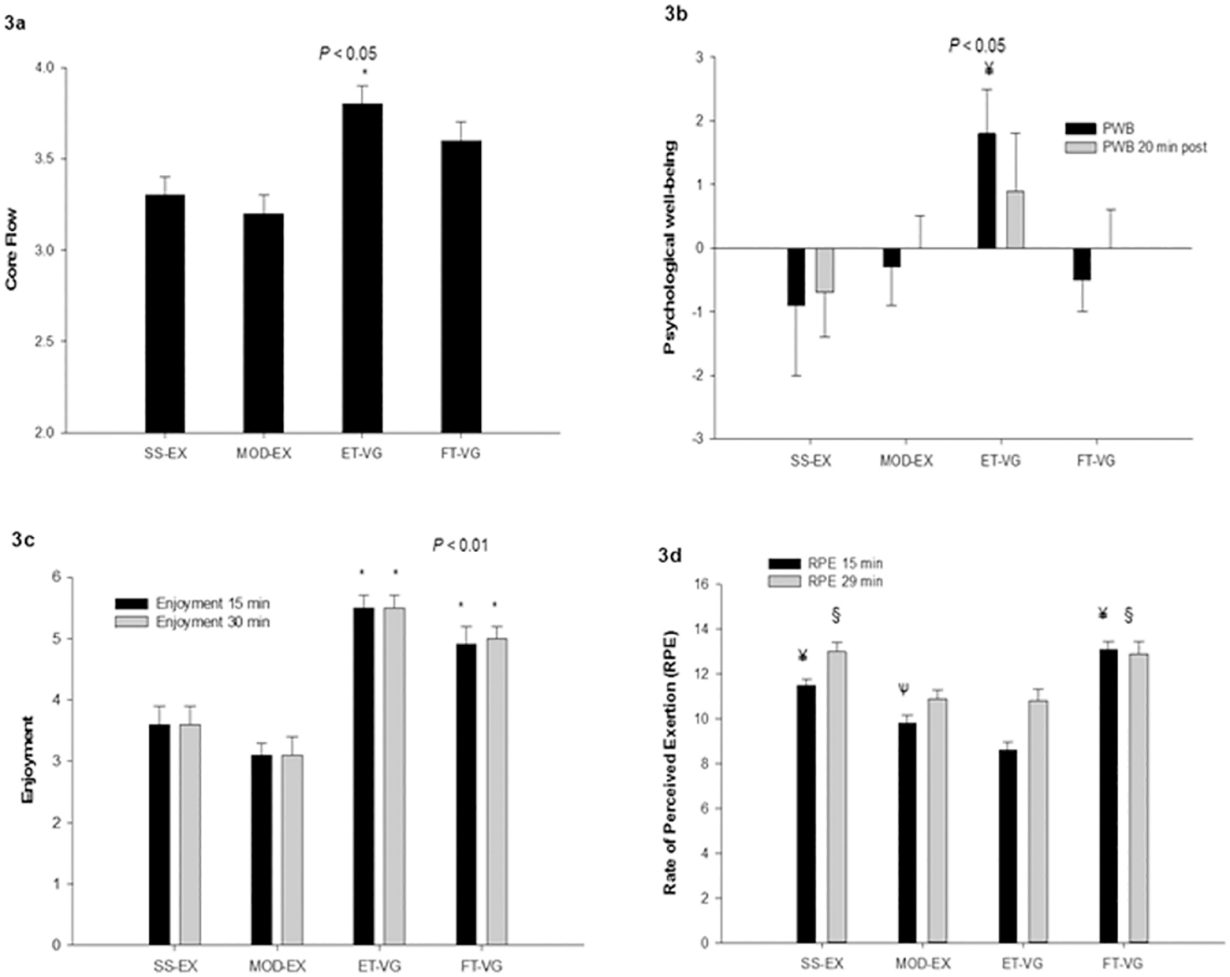
**A.** State of core flow immediately after the trials. All data presented as mean ± SEM. *significantly different than SS-EX and MOD-EX, *P* < 0.05. **b** Differences in psychological well-being before and immediately after the trials (PWB post diff) and before and 20 min after trial completion (PWB 20 min post diff). ¥ significantly different than all other trials, *P* < 0.05. **c** Enjoyment at 15 and 29 min, and average scores. *significantly different than SS-EX and MOD-EX, *P* < 0.01. **d.** Rate of perceived exertion (RPE) at 15 and 29 min and average scores. ¥ significantly different than all other trials, *P* < 0.05; ѱ significantly different than ET-VG, *P* < 0.05;.§ significantly different than MOD-EX and ET-VG, *P* < 0.01. All data presented as mean ± SEM.

We report a significant effect of trial on the core flow (p < 0.05), η^2^ = 0.520, with ET-VG resulting in higher states of flow than both exercise trials (Fig 3A).

There were no differences in baseline scores of psychological well-being but there is an effect of trial (p < 0.05), η^2^ = 0.129, with ET-VG resulting in significantly higher psychological wellbeing values immediately after the trial (Fig 3B). There was a significant effect of sex (p < 0.05), partial η^2^ = 0.257, with men reporting higher psychological well-being during and immediately after each trial, except for the FT-VG. For women, well-being decreased immediately after the SS-EX and MOD-EX trials, but increased after the ET-VG and remained the same after the FT-VG trial.

The gaming trials resulted in significantly greater enjoyment ratings than the exercise trials (p < 0.001), η^2^ = 0.810 (Fig 3C). No significant differences were found in enjoyment ratings between the exercise trials or between the gaming trials. Mean enjoyment did not change over time in any of the trials (p < 0.642) or between men and women.

We found a significant relationship between average enjoyment during the trial and %VT in the SS-EX trial only (p< 0.01). In this trial, 31.9% of enjoyment could be accounted for the variability in intensity relative to the ventilatory threshold.

Repeated measures ANOVA revealed a significant effect for the condition on RPE levels (*P* < 0.001, partial η^2^ = 0.654) as seen in Fig 3D. The SS-EX trial had higher average RPE ratings than MOD-EX (p < 0.001) and ET-VG (p < 0.001), but there were no differences compared to the FT-VG trial. The FT-VG trial also had significantly higher RPE values than both MOD-EX and ET-VG (p < 0.001).

## Discussion

The main finding of this study was that active video games can meet the criteria for moderate-to-vigorous physical activity and lead to more positive affective judgements than conventional exercise trials. These data indicate that active video games provide an adequate stimulus to meet the physical activity guidelines and could form part of future physical activity recommendations.

The ET-VG and FT-VG trials in this study resulted in 5.1±0.3 and 6.4±0.2 METs, which qualify them as moderate and vigorous intensity exercise, respectively. These results are higher than those reported in other cross-sectional studies[18, 19, 35] and the differences may be explained by variation in the difficulty level of the game. There is a wide variety of active video games on the market and the variation in the design and effort required makes it difficult to compare outcomes or reach consensus about the efficacy of this form of activity. Other studies that used different FT-VG such as the Wii Fit, report a range of physiological outcomes[19, 29, 36, 37]. Miyachi et al[36] reported lower MET values for resistance (1.7–5.6 METs) and aerobic (2.7–5.1 METs) in a metabolic chamber compared to the 6.3 and 6.5 METs, respectively, in the present study.

In other cases, fitness-themed video games focus on light aerobic and balance games[19]. These games may be targeted at older adults and require finer motor control but the rate of energy expenditure is likely to be lower than in resistance activities. It is also possible that the initial physical activity or fitness levels may also be a contributing factor. Our participants were in the 60th percentile of the ACSM normative values for $\dot{V}O_{2}\max$[38] and it is possible that active video game play may provide a greater physiological stimulus to inactive individuals compared to more aerobically fit participants. Finally, the participants’ motivation and experience playing the active video games is likely to have an impact on the results[39].

The SS-EX trial resulted in the highest exercise intensity, attaining the criteria for vigorous exercise based on heart rate, oxygen consumption and energy expenditure criteria[26]. Similar values have been reported in the literature in studies with young, normal weight participants[8] but the MET values are higher than those reported for walking/jogging in the literature(3). However, the SS-EX trial had significantly lower enjoyment than both AVG trials and lower psychological well-being than the ET-VG trial.

During the SS-EX trial, participants were <2% below their ventilatory threshold but there was a high inter-individual variability in the selected exercise intensity, consistent with other studies[40]. However, the greater autonomy over the exercise intensity did not result in greater enjoyment or flow scores as would have been expected[8]. In this study, participants may have selected the intensity at the upper range for normal exercise and this might have a negative effect on enjoyment and flow.

Our results are similar to those in the literature reporting that active video games result in moderate exercise intensities. Participants enjoyed the two active video game trials significantly more than the two conventional exercise trials but it was interesting to note some differences between the two video game trials. The FT-VG used in this study resulted in a higher % HRmax than the MOD-EX and ET-VG trials, and was more enjoyable than both exercise trials. This fitness themed game could be described as vigorous based on the heart rate max criteria. On the other hand the ET-VG was moderate intensity exercise and had the most positive affect states. The percentage $\dot{V}O_{2}\max$ and rate of energy expenditure during the ET-VG were significantly lower than the other trials. We could only find one other study[35] that used Kinect Adventures but there are significant differences in the design of both studies and a direct comparison is not possible. It will be important to address the issue of data harmonisation from different video game studies to better understand the impact on physiological outcomes. Addressing these issues will be very important to provide more definitive evidence that active video games can be used to attain the physical activity recommendations.

The four trials tested in this study resulted in very different affect states and psychological well-being that might have important implications for exercise prescription. The highest enjoyment ratings occurred in the active video game trials, where subjects did not experience the autonomy to decide on games and intensity levels contradicting some evidence in the literature[8]. A possible explanation is that participants experienced more positive psychological and affect states during the active video game trials as the higher score for core flow and psychological well-being during the ET-VG trial shows. This increased state of flow and the enhanced sense of well-being might have compensated for the lack of autonomy during the active video game trials. While video games and sport are two domains in which an enhanced state of flow is likely to occur[41], there is a lack of research on active video games and the state of flow. Thin et al[42] compared the flow state and enjoyment of 14 young adults while playing 6 different active video games and cycling exercise and found that two of the flow dimensions (challenge-skill balance and merging of action and awareness) scored significantly higher than the exercise trial. We did not analyse the 9 different dimensions of flow separately, but got a significant difference in the overall state of flow.

The findings of this study partly supports the principles of the dual-mode model theory[13]. The highest enjoyment ratings occurred in the ET-VG trial, when the participants were working at the lowest intensity in relation to individuals’ ventilatory threshold. However, the FT-VG and the MOD-EX trials resulted in similar percentages of ventilatory threshold, but enjoyment ratings were significantly higher during the FV-TG than during the MOD-EX. The complex interplay of cortically mediated cognitive processes and the physiological changes leading to increased metabolic stress were modified during the FV-TG trial and as result of this enjoyment ratings were higher than during MOD-EX. We also found that ~30% of enjoyment during the SS-EX trial could be accounted for the variability in intensity in relation to the ventilatory threshold but this was not evident in the other trials. It is clear that other factors apart from exercise intensity were playing a role in determining the affect response to the exercise and games. One example is RPE, which was similar in the FT-VG and SS-EX trials and significantly greater than the MOD-EX and ET-VG.

We acknowledge that certain aspects of the design and implementation may limit the interpretation of our findings. Firstly, using an average of the last 3 min of the 10 min data collection at rest prior to each trial as the baseline measures might not be enough to get the participants in a complete steady-state for the physiological parameters of interest (HR,$\dot{V}O_{2}$ and rates of EE). A longer rest period would have resulted in more accurate baselines measures. Secondly, the 3-hr lapse since last eating should be sufficient to allow for digestion and absorption of a light meal but there may be a residual effect. The research team strove to ensure the data was reliable by performing the tests at the same time of day and following the same protocol for all subjects while trying to maintain a ‘real life’ study design. Thirdly, this study examined the affective responses to each single bout of either exercise or active video game. While affective responses to a single session are useful, there is a need for intervention studies to examine the long term effects. Finally, sixteen participants in this study were normal weight, four were overweight and four were classified as obese. We did not examine the effect of body weight in the different physiological and psychological parameters due to low numbers of overweight and obese participants. The results obtained could be different in a cohort of mainly overweight or obese participants.

## Conclusion

There are a variety of AVGs on the market and our results cannot be generalised. The AVGs used in this study resulted in moderate exercise intensities that comply with intensity recommendations of the ACSM physical activity guidelines. The fitness-themed video game yielded a similar EE and rate of EE to moderate intensity exercise, but lower than self-selected exercise. The AVG trials resulted in the greatest affect states and psychological well-being of all conditions. Participants rated the entertainment-themed video games as the most enjoyable and experienced a higher state of flow and psychological well-being during this condition than during the exercise trials.

The results of this study have implications in the fight against adult physical inactivity and sedentary behaviour. Video game usage is widely extended among the population, and this study shows that AVG technology can be a useful and efficient tool to help people to meet the PA recommendations and to reduce sedentary behaviour. The fitness-themed video game resulted in high exercise intensity but was not as enjoyable as the entertainment-themed video game. Video game designers could take account of this and try to introduce elements in the games that make them more attractive, even if this means making them less demanding from a physiological point of view.

## Supporting information

S1 Appendix Active video games (AVGs) used in the study, play time and level played. (DOCX)
