# Supplementary material for: Energy expenditure and affect responses to different types of active video game and exercise
Source: PLoS One. 2017 May 1;12(5):e0176213. doi: 10.1371/journal.pone.0176213 (PMC5411095; doi:10.1371/journal.pone.0176213)
Supplement: S1 Appendix — (DOCX) [file pone.0176213.s001.docx]

**Supporting information**

Appendix 1 Active video games (AVGs) used in the study, play time and level played.

|  | **Game** | **Player Activity** | **Times played and level** |
| --- | --- | --- | --- |
| **Entertainment- Themed Video Games**  **(ET-VG)** | River Rush | Control a raft going down a river using your body | 10 min  3 times at advanced |
|  | Rally Ball | Use your body to deflect balls and hit them down the court to destroy targets at the end of the lane | 10 min  Once at intermediate  Twice at advanced |
|  | Reflex Ridge | Race through a rollercoaster-like track full of obstacles and dodge the hazards. | 10 min  Once at intermediate  Twice at advanced |
| **Fitness- Themed Video Games**  **(FT-VG)** | Wall Breaker | Hit ice blocks to break them and dodge a hammer | 4 min  Once at advanced |
|  | Run the world | A virtual jog through the streets of New York | 4 min  Once at advanced |
|  | Jump Rope | Syncing steps with virtual footprints | 6 min  Once at advanced  Once at extreme |
|  | Cardio  (Legs, Burn  & Shape D) | Variety of dynamic exercises that involve coordination of upper and lower body. | 16 min  Only available level |

Entertainment-themed video game (ET-VG) included river rush, rally ball and reflex ridge. Fitness-themed video game (FT-VG) included wall breaker, run the world, jump rope and cardio (legs, burn & shape D).
